# Supplementary material for: Serum Cytokine Profile, Beta-Hexosaminidase A Enzymatic Activity and GM2 Ganglioside Levels in the Plasma of a Tay-Sachs Disease Patient after Cord Blood Cell Transplantation and Curcumin Administration: A Case Report
Source: Life (Basel). 2021 Sep 24;11(10):1007. doi: 10.3390/life11101007 (PMC8539434; doi:10.3390/life11101007)
Supplement: Supplementary file 1 [file life-11-01007-s001.zip › life-1354773-supplementary.pdf]

Supplementary Material

Table S1. Multiplex analysis of cytokines in the blood serum of a patient with TSD.

Analyte	Analyte concentration in blood serum. pg/ml									
		Patient								
	Control (n = 10)	Day 0 Before UCBCT	Day 184 After UCBCT	Day 252 500 mg/day	Day 262 1000 mg/day	Day 276 3500 mg/day	Day 290 3500 mg/day	Day 305 3500 mg/day	Day 323 3500 mg/day	Day 339 3500 mg/day
EGF	111.6 ± 103.4	345.4 ± 0.8	153.4 ± 16.9	151.4 ± 38.21	263.6 ± 17.2	260.4 ± 4.9	63.2 ± 64.3	180.5 ± 31.4	514.6 ± 56.6	184.5 ± 12.4
Eotaxin	64.8 ± 28.7	200.6 ± 1.9	155.7 ± 8.4	192.0 ± 13.3	203.8 ± 2.4	160.8 ± 5.6	4.1 ± 0.9	146.4 ± 20.4	107.5 ± 4.3	93.1 ± 17.5
FGF-2	86.81 ± 101.17	37.02 ± 4.60	30.46 ± 8.51	22.10 ± 8.07	23.64 ± 10.25	39.22 ± 9.72	18.55 ± 3.05	140.9	40.15 ± 5.20	34.45 ± 0.97
FLT-3	15.94 ± 16.27	3.95 ± 0.23	2.12 ± 1.77	0.24 ± 0.24	3.58 ± 0.89	3.38 ± 1.49	0.75 ± 0.75	17.44 ± 17.44	8.14 ± 1.36	10.27 ± 0.75
Fractalkine	87.58 ± 60.63	38.49 ± 0.00	35.52	13.86	29.01	55.12	35.52	157.31 ± 185.96	53.70 ± 21.51	57.02 ± 6.97
G-CSF	40.35 ± 24.78	16.90 ± 0.37	13.04 ± 10.92	8.92 ± 2.47	11.77 ± 2.36	19.14 ± 6.53	13.17 ± 0.39	47.31 ± 51.02	18.20 ± 0.74	13.96 ± 2.29
GM-CSF	26.92 ± 15.24	14.61 ± 1.32	11.71 ± 4.98	10.95 ± 4.74	11.99 ± 1.51	16.49 ± 4.84	11.53 ± 0.44	38.64 ± 37.03	15.88 ± 5.71	15.23 ± 2.19
GRO	1067.05 ± 730.84	1851.31 ± 148.93	1403.99 ± 415.19	1491.48 ± 210.49	1385.85 ± 442.97	1385.64 ± 32.82	702.75 ± 85.92	1506.28 ± 152.78	988.86 ± 1.28	1123.12 ± 72.65
IFN-γ	36.94 ± 51.24	10.71 ± 0.40	7.51 ± 1.68	7.21 ± 1.26	8.54 ± 1.03	12.1 ± 4.00	5.41 ± 0.43	29.25 ± 30.74	10.98 ± 2.42	12.69 ± 1.60
IL-10	4.83 ± 2.21	3.10 ± 0.23	2.76 ± 1.03	2.50 ± 0.30	2.88 ± 0.15	3.90 ± 0.52	3.26 ± 0.31	8.76 ± 7.77	6.05 ± 0.86	5.80 ± 0.51
IL-12 (p40)	37.48 ± 36.83	<1.31	1.31	<1.31	<1.31	4.94	<1.31	97.84	3.19	<1.31
IL-12 (p70)	9.22 ± 12.30	3.45 ± 0.16	2.53 ± 1.13	2.45 ± 0.30	2.68 ± 0.62	4.00 ± 1.26	3.22 ± 0.16	15.10 ± 17.59	3.34 ± 0.65	2.89 ± 0.32
IL-13	8.87 ± 6.14	2.06 ± 0.00	2.62	<0.44	1.17 ± 0.72	2.37 ± 2.12	1.28	23.95	2.97 ± 1.28	1.47 ± 0.84
IL-15	5.32 ± 4.47	2.18 ± 0.14	1.35 ± 1.04	1.33 ± 0.26	1.61 ± 0.54	2.88 ± 1.12	2.18 ± 0.41	10.96 ± 13.36	2.34 ± 0.90	2.18 ± 0.41
IL-17A	9.67 10.84	5.41 0.15	5.02 1.31	4.67 0.30	5.33 0.78	5.81 1.01	5.62 0.00	8.66 5.93	6.68 0.90	5.94 0.97
IL-1Rα	133.00 ± 181.83	24.64 ± 3.56	14.30 ± 11.06	8.58 ± 4.33	14.69 ± 8.78	26.42 ± 16.18	25.25 ± 0.90	149.81 ± 202.69	21.07 ± 8.61	20.96 ± 5.17

Analyte	Analyte concentration in blood serum. pg/ml									
		Patient								
	**Control (n = 10)**	**Day 0 Before UCBCT**	**Day 184 After UCBCT**	**Day 252 500 mg/day**	**Day 262 1000 mg/day**	**Day 276 3500 mg/day**	**Day 290 3500 mg/day**	**Day 305 3500 mg/day**	**Day 323 3500 mg/day**	**Day 339 3500 mg/day**
IL-1α	55.24 ± 60.18	<3.2	<3.2	<3.2	<3.2	20.82	147.67	<3.2	<3.2	<3.2
IL-1β	90.02 ± 195.09	2.29 ± 0.00	1.84 ± 0.32	1.73 ± 0.16	2.01 ± 0.40	2.61 ± 0.69	1.92 ± 0.28	6.46 ± 6.53	2.32 ± 0.52	2.35 ± 0.40
IL-2	5.81 ± 6.31	3.35 ± 0.12	2.81 ± 0.49	2.49 ± 0.04	2.46 ± 0.16	3.52 ± 0.93	2.78 ± 0.62	6.87 ± 6.41	2.92 ± 0.33	3.21 ± 0.25
IL-3	3.90 ± 2.80	2.74 ± 0.20	2.48 ± 0.95	2.03 ± 0.06	2.25 ± 0.25	3.35 ± 0.93	2.52 ± 0.52	11.12 ± 12.17	2.97 ± 0.40	2.33 ± 0.00
IL-4	26.18 ± 26.97	8.79 ± 2.71	1.84	<0.66	3.01 ± 0.00	5.70 ± 6.00	31.13	58.81	5.33	3.01
IL-5	2.30 ± 2.14	1.29 ± 0.01	1.18 ± 0.30	1.20 ± 0.18	1.23 ± 0.07	1.58 ± 0.30	1.55 ± 0.12	2.91 ± 2.54	1.42 ± 0.16	1.28 ± 0.04
IL-6	66.49 ± 108.84	<0.55	<0.55	<0.55	<0.55	<0.55	<0.55	8.37	<0.55	<0.55
IL-7	7.26 ± 3.78	4.17 ± 0.15	4.46 ± 0.47	4.11 ± 0.78	4.40 ± 0.18	5.19 ± 0.83	3.14 ± 1.50	7.97 ± 5.72	4.50 ± 0.23	4.92 ± 0.45
IL-8	7.8 ± 6.0	21.30 ± 1.26	8.11 ± 0.19	7.18 ± 1.12	8.88 ± 1.68	9.74 ± 0.08	4.84 ± 0.48	11.56 ± 3.91	69.58 ± 9.31	9.33 ± 0.52
IL-9	2.14 ± 1.00	1.95 ± 0.19	1.42 ± 0.31	1.37 ± 0.16	1.44 ± 0.25	1.95 ± 0.43	1.01 ± 0.56	3.39 ± 3.09	1.84 ± 0.28	1.84 ± 0.04
IP-10	73.9 ± 26.3	80.37 ± 2.69	95.22 ± 34.16	35.22 ± 2.52	66.13 ± 13.82	59.49 ± 6.46	71.40 ± 6.72	62.06 ± 18.66	23.35 ± 1.12	28.82 ± 2.32
IFN-α2	71.29 ± 52.71	52.21 ± 2.99	30.33 ± 24.88	28.02 ± 11.75	25.75 ± 0.00	60.83 ± 18.25	38.47 ± 10.23	153.15± 165.22	43.21 ± 9.74	41.13 ± 0.00
MCP-1	498.76 ± 313.57	495.64 ± 30.47	376.06 ± 70.66	414.49 ± 25.09	350.89 ± 10.61	431.35 ± 14.22	317.76 ± 3.05	418.13 ± 4.43	472.58 ± 12.83	459.27 ± 24.08
MCP-3	81.19 ± 110.50	<2.6	<2.6	<2.6	<2.6	<2.6	<2.6	29.75	<2.6	<2.6
MDC	413.3 ± 175.8	1127.93 ± 5.67	874.83 ± 264.53	836.22 ± 106.58	542.88 ± 28.79	753.40 ± 60.32	272.61 ± 284.41	832.64 ± 105.23	756.08 ± 95.04	1052.10 ± 23.14
MIP-1α	480.81 ± 818.68	3.17 ± 0.00	2.97 ± 0.38	2.77 ± 0.15	2.71 ± 0.23	3.31 ± 0.30	2.95 ± 0.11	5.09 ± 2.93	3.37 ± 0.03	3.02 ± 0.21
MIP-1β	265.97 ± 530.85	17.57 ± 0.82	11.24 ± 4.74	11.24 ± 4.74	15.79 ± 1.70	21.93 ± 5.34	8.78 ± 4.64	46.90 ± 47.44	21.93 ± 5.34	20.39 ± 1.56
PDGF-AA	1666.50 ± 867.94	2715.05 ± 74.53	1506.22 ± 1296.07	1228.69 ± 214.92	2014.0 ± 193.58	2106.62 ± 179.44	291.79 ± 243.00	1481.40 ± 208.94	668.78 ± 33.70	577.02 ± 30.00

Analyte	Analyte concentration in blood serum. pg/ml									
		Patient								
	Control (n = 10)	Day 0 Before UCBCT	Day 184 After UCBCT	Day 252 500 mg/day	Day 262 1000 mg/day	Day 276 3500 mg/day	Day 290 3500 mg/day	Day 305 3500 mg/day	Day 323 3500 mg/day	Day 339 3500 mg/day
PDGF-AB/BB	13383.03 ± 5559.26	18810.22 ± 79.35	13559.09± 6397.54	13709.74± 3150.71	16717.45 ± 465.17	17081.77 ± 221.58	7567.23 ± 496.18	13494.22 ± 3767.37	16254.75 ± 976.86	17076.81 ± 463.01
RANTES	1527.08 ± 1796.76	900.40 ± 6.02	721.65 ± 295.42	533.02 ± 207.27	730.69 ± 54.96	837.43 ± 3.40	463.61 ± 77.27	440.44 ± 200.50	828.72 ± 0.32	797.66 ± 76.37
TGF-α	6.14 ± 4.97	4.88 ± 0.04	6.17 ± 1.07	5.80 ± 1.26	8.58 ± 0.40	7.56 ± 0.04	6.27 ± 0.13	7.61 ± 2.38	7.80 ± 0.54	6.09 ± 0.24
TNFα	59.43 ± 84.87	15.77 ± 0.93	12.92 ± 1.74	9.06 ± 2.50	12.09 ± 0.31	14.41 ± 0.13	12.40 ± 1.00	15.95 ± 11.75	15.03 ± 3.09	16.69 ± 0.37
TNFβ	1.77 ± 3.08	<0.14	<0.14	<0.14	<0.14	0.56 0.12	13.72	<0.14	<0.14	<0.14
VEGF	138.21 ± 133.74	387.52 ± 29.81	346.73 ± 10.09	299.56 ± 60.66	298.64 ± 33.30	335.58 ± 0.41	151.65 ± 82.09	327.34 ± 51.23	296.08 ± 3.72	305.02 ± 15.05
sCD40L	4576.1 ± 3168.7	10260.30 ± 793.15	6275.93 ± 2162.01	6755.18 ± 1381.52	8371.31 ± 542.94	8683.64 ± 11.13	4436.18 ± 702.71	6369.35 ± 1405.86	8264.04 ± 110.10	8345.55 ± 393.17

Table S2. Results of the clinical studies of curcumin.

Study participants	Drug	Dose (mg)	Duration	Blood concentration (ng/mL)	Half-life (hours)	Effect	Reference
Healthy volunteers	γ-cyclodextrin curcumin formulation	10 000	Single dose administration	73.2	6.8 ± 0.8	The γ-cyclodextrin-curcumin composition significantly improves the absorption of curcuminoids	[76]
Healthy volunteers	Curcumin nanoparticles	150	Single dose administration	189	9.7 ± 2.1	No side effects were observed	[77]
Healthy volunteers	Lipid nanoparticles	160	Single dose administration	22.43	7.5 ± 2.4	No side effects were observed	[78]
Patients diagnosed with osteosarcoma	Lipid nanoparticles	2000	Single dose administration	32.51	2.45 ± 0.47	No side effects were observed	[78]
Patients diagnosed with osteosarcoma	Lipid nanoparticles	3000	Single dose administration	31.42	7.50 ± 0.01	No side effects were observed	[78]

Patients diagnosed with osteosarcoma	Lipid nanoparticles	4000	Single dose administration	41.15	7.62± 1.09	No side effects were observed	[78]
Patients diagnosed with cancer	Curcumin (diferuloylmethane) 99.3%	8 000	3 months	652.03		No dose-related toxicity was observed	[79]
Patients diagnosed with pancreatic cancer	Theracurmin®	400	9 months	440		No side effects were observed	[80]
Older people	Longvida®	400	4 weeks	2575	2.8	Cognitive function improvement	[81]
Healthy volunteers	85% curcumin. 12% demethoxycurcumin. and 3% bisdemethoxycurcumin	2 000	Single dose administration	11		No side effects were observed	[82]
Healthy volunteers	C3 Complex™	12 000	Single dose administration	1730	6.77	Curcumin glucuronide and curcumin sulfate were found in plasma after oral administration	[83]
Healthy volunteers	Theracurmin®	90 (twice a day)	18 months			Memory and attention improvement	[84]
Patients diagnosed with knee osteoarthritis	BCM-95®	500 (three times a day)	28 days			Similar efficacy to diclofenac	[85]
